# Implementation Cascade of a Social Network–Based HIV Self-testing Approach for Men Who Have Sex With Men: Cross-sectional Study

**DOI:** 10.2196/46514

**Published:** 2023-04-26

**Authors:** Tsz Ho Kwan, Denise Pui Chung Chan, Samuel Yeung-shan Wong, Shui Shan Lee

**Affiliations:** 1 Stanley Ho Centre for Emerging Infectious Diseases The Chinese University of Hong Kong Hong Kong China (Hong Kong); 2 Jockey Club School of Public Health and Primary Care The Chinese University of Hong Kong Hong Kong China (Hong Kong)

**Keywords:** community-based testing service, cross-sectional study, HIV self-test, HIV testing, HIV, implementation cascade, men who have sex with men, social network, virus transmission

## Abstract

**Background:**

HIV testing is the cornerstone of strategies for achieving the fast-track target to end the AIDS epidemic by 2030. Self-testing has been proven to be an effective health intervention for men who have sex with men (MSM). While social network–based approaches for distributing HIV self-tests are recommended by the World Health Organization, their implementation consists of multiple steps that need to be properly evaluated.

**Objective:**

This study aimed to assess the implementation cascade of a social network–based HIV self-test approach for reaching MSM who had never undergone testing in Hong Kong.

**Methods:**

This is a cross-sectional study. Seed MSM participants were recruited through different web-based channels, who in turn invited their peers to participate in this study. A web-based platform was set up to support the recruitment and referral process. Participants could request for an oral fluid or a finger-prick HIV self-test, with or without real-time support, after completing a self-administered questionnaire. Referrals could be made upon uploading the test result and passing the web-based training. Characteristics of participants completing each of these steps and their preferences for the type of HIV self-test were evaluated.

**Results:**

A total of 463 MSM were recruited, including 150 seeds. Participants recruited by seeds were less likely to have previously been tested for HIV (odds ratio [OR] 1.80, 95% CI 1.06-3.04, *P*=.03) and have lower confidence in performing self-tests (OR 0.66, 95% CI 0.45-0.99, *P*=.045). Almost all (434/442, 98%) MSM who completed the questionnaire requested a self-test, of whom 82% (354/434) had uploaded their test results. Participants requesting support were new to self-testing (OR 3.65, 95% CI 2.10-6.35, *P*<.001) and less confident in carrying out the self-test correctly (OR 0.35, 95% CI 0.22-0.56, *P*<.001). More than half (216/354, 61%) of the eligible participants initiated the referral process by attempting the web-based training with a passing rate of 93% (200/216). They were more likely to have sought sex partners (OR 2.20, 95% CI 1.14-4.25, *P*=.02), especially through location-based networking apps (OR 2.13, 95% CI 1.31-3.49, *P*=.002). They also gave higher usability scores along the implementation cascade (median 81 vs 75, *P*=.003).

**Conclusions:**

The social network approach was effective in diffusing HIV self-tests in the MSM community and reaching nontesters. Support and option to choose a preferable type of self-test are essential to address users’ individual needs when delivering HIV self-tests. A positive user experience throughout the processes along the implementation cascade is vital to transform a tester into a promoter.

**Trial Registration:**

ClinicalTrials.gov NCT04379206; https://clinicaltrials.gov/ct2/show/NCT04379206

## Introduction

Globally, the incidence of HIV infections has been decreasing over the last decades. However, men who have sex with men (MSM) remained the driving force of virus transmission in Western Europe and North America [1], the prevalence of which was 16% in sub-Saharan Africa [2] and as high as 28% in Asia [3]. To end the global epidemic, the Joint United Nations Programme on HIV/AIDS (UNAIDS) has promoted the fast-track target, that is, by the year of 2030, 95% people living with HIV are aware of their HIV status, 95% people knowing their status receive antiretroviral treatment, and 95% people receiving treatment achieve viral suppression [4]. To achieve the “first 95” goal, HIV testing plays a critical role. However, HIV testing rate in the community has been suboptimal. The ever-testing rate among MSM ranged from 52% in Brazil [5], 70% in China [6], and 85% in the United States [7]. In Hong Kong, where MSM accounted for a majority of the newly diagnosed HIV cases [8] and pre-exposure prophylaxis (PrEP) is only available from clinical studies but not public health care services [9,10], only 64% had ever tested for HIV and half (50%) of the population last tested within a year [11]. Various reasons were cited as barriers to HIV testing in the community, such as low perceived risk and poverty [11,12]. Even though free and accessible voluntary counseling and testing services were offered, concerns of stigma, confidentiality, and privacy issues could still be obstacles for some MSM to access HIV services [12,13]. With the development of HIV self-test products, it is possible to get tested in a private, confidential environment at a time and place convenient to the user. Results from a meta-analysis showed that HIV self-testing could increase test uptake and the frequency of testing [14]. It could also contribute to the identification of more undiagnosed HIV infections for linking them to care compared with standard HIV testing approaches.

For MSM, HIV self-testing is a multistep procedure beginning with the provision of test kits, continuing with the performance of the tests, and ending with the collection of test results for linking those who tested positive to follow-up services. Effective strategies for distributing HIV self-tests to community members are vital, especially for reaching those who are reluctant to attend sexual health services. A previous study found that both monetary incentives and peer referral could enhance the secondary distribution of HIV self-tests [15]. Social network–based approaches are recommended by the World Health Organization as part of a comprehensive package of HIV prevention [16]. In order to support the referral process and empower participants to invite their peers to receive HIV testing, training on HIV knowledge, self-test kit use, and resources for follow-up confirmatory testing would be important [17-19]. The desire to help fellow community members and usability of the digital referral system are the cornerstones that enabled MSM to make referrals [19,20]. To ensure the robustness of the approach and the quality of self-test results, monitoring and evaluation along the steps would be crucial. The UNAIDS issued an operational guidance on using a cascade approach to evaluate an HIV prevention intervention [21]. A previous study has similarly applied the cascade to the implementation of PrEP in the United States [22]. Against these backgrounds, this study was conceptualized to determine the feasibility of a social network–based HIV self-test promotion program with an implementation cascade framework and its potential in reaching harder-to-reach MSM, particularly those who had never been HIV-tested.

## Methods

### Study Design and Implementation Cascade

**Figure 1:**
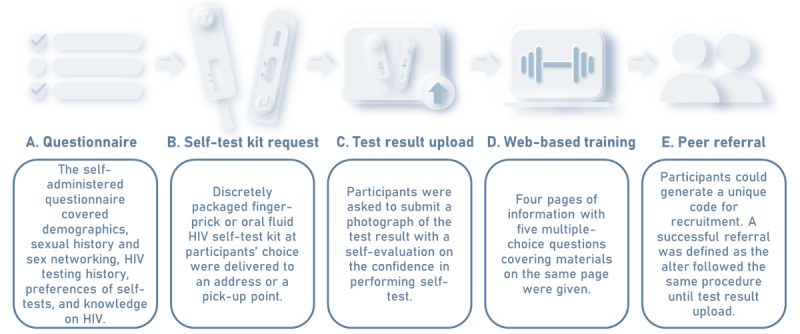
Implementation cascade of the social network–based HIV self-test study.

A cross-sectional study design was adopted. Seed participants were recruited from various internet platforms, including social media platforms, a web-based forum, and location-based networking apps used by MSM. The implementation cascade in this study consisted of the following 5 steps: enrollment with questionnaire completion, self-test kit request, test result upload, web-based training, and peer referral (Figure 1). One could only complete the steps in 1 direction through the bilingual (English and Chinese) web-based platform after registration and giving web-based consent. Upon completing a self-administered, web-based questionnaire after enrollment, participants could request a discretely packaged HIV self-test kit with the choice of either a finger-prick or oral fluid test, which was delivered without charge. They could also ask for real-time support, including in-person, video call, and instant messaging support, at kit request. Participants requesting real-time support received a text message through instant messaging apps to schedule a time for the self-test. A support hotline was available to all study subjects. Participants were asked to submit a photograph of the test result after performing the self-test, followed by self-evaluation on the confidence of performing the self-test correctly on a scale of 1 to 10. To encourage return, an incentive of HK $20 (US $2.56) was given after manual verification of the result. Participants returning positive or invalid results were invited for retests by venipuncture at the research center in a hospital. Referral to HIV services was made upon consent if it were confirmed positive by the government laboratory.

Peer referral formed the subsequent set of activities following self-testing in the study. A web-based training session was designed at the prerequisite stage of the referral process. It consisted of 4 pages of basic information on HIV, HIV self-tests, and study logistics, with 5 multiple-choice questions covering materials on the same page. Out of 20 questions, participants needed to give 14 correct answers to proceed. There was no limit on the number of attempts until pass. Correct responses to the questions with wrong answers were given after the passing attempt. Participants could then create a unique referral code for each peer he wished to recruit after completing a short questionnaire on the relationship between the participant and the referee, his testing and condom use practices, and reason for referral. In order to prevent arbitrary distribution of referral codes, which may jeopardize data integrity, the year of birth inputted in the referral form must match, with an error margin of 1, with the one completed by the referee in the questionnaire. The referee could register on the same platform and go through the same steps described above. This referral procedure carried the benefit of collecting information about the peers invited by the study participants but who eventually did not join this study, addressing a common limitation of other social network–based referral studies [20].

### Subjects

Eligibility criteria included being male, aged 18 years or above, ever had sex with men, able to communicate in written English or Chinese, residence in Hong Kong, and referral by a peer or research team. A sample size of 384 provided a 95% confidence level and 5% CI for an unlimited population size with maximized uncertainty given a 50% response distribution. The target sample size was set at 400. With a preliminary estimate of a seed-to-alter ratio of about 5 [18], about 64 MSM were planned to be recruited as seeds from the community for promoting HIV self-testing in the first round. An additional round of seed recruitment was made 1 month apart in order to reach the targeted number of subjects.

### Questionnaire and Assessments

The questionnaire consisted of the following 6 parts: demographics, history of sexual behaviors, sexual history and sex networking in the past year, HIV testing history, preferences for self-tests, and knowledge of HIV. Demographic variables included ethnic identity, education level, employment status, monthly income, and age. Sex networking patterns at different physical and web-based channels in the past year were separately inquired. Sexual behaviors in the preceding year included condom use with newly and previously met regular and nonregular partners, history of chemsex, group sex, PrEP use, and perception of HIV and sexually transmitted infection (STI) risk. On HIV self-testing, items included preference of blood or oral fluid sampling, confidence in collecting sufficient sample for self-test and interpreting results correctly on a scale of 1 to 10, preference of location for HIV testing, consideration factors regarding the place for receiving an HIV test and an HIV self-test product, and preferred modes of HIV self-test support. To assess user experience with HIV self-testing, a single ease question, a 7-point scale assessing the difficulty of the task, was displayed to collect users’ responses. After uploading the self-test result and making the first referral, the system usability scale with 10 questions was applied. The system usability score was computed within the range of 0 to 100.

### Statistical Analysis

To echo the study objectives, the primary outcome was the characteristics of participants recruited by the seeds, in particular the proportion of never HIV testers. Referring to the implementation cascade, secondary outcome measures include the proportion of MSM not having tested for HIV recently, the preferences of the 2 types of HIV self-test kits, proportion and characteristics of participants requiring support in performing HIV self-test, proportion and characteristics of participants who uploaded their HIV self-test result, proportion and characteristics of participants who were willing to recruit their peers in getting HIV self-tested through this study, and number and proportion of HIV positive results reported by the participants. A chi-square test was used to compare between seeds and participants recruited by them (alters) and to identify participants who subsequently returned their HIV self-test results and attempted training as a surrogate of interest in referring peers to get self-tested in the implementation cascade. Preferences for the types of HIV self-tests and support were similarly assessed. Continuous and categorical variables were analyzed using the Mann-Whitney *U* test and the chi-square test, respectively. Multivariable logistic regression with both backward and forward selection using variables with a significance of *P*<.10 was used to determine predictors for participants’ preferences in types of self-tests and support. A *P* value of <.05 was considered significant. The electronic questionnaire system checked for missing inputs before allowing response submission; therefore, there were no missing data in the questionnaire. As per the nature of an implementation cascade, some data were unavailable for participants who never achieved a particular stage, in which case only subgroup analyses were performed. All analyses were conducted using R (The R Foundation).

### Ethical Considerations

This study was approved by Joint Chinese University of Hong Kong—New Territories East Cluster Clinical Research Ethics Committee (CREC Ref. No. 2020.087), registered at ClinicalTrials.gov (identifier NCT04379206), and conducted in accordance with the Declaration of Helsinki. Web-based informed consent was obtained from all individual participants included in the study. The conduct of the study was in compliance with the requirements of the Personal Data (Privacy) Ordinance. Participants were awarded an HK $20 (US $2.56) incentive for each successful referral made, as defined by referee’s test result upload. One could receive up to HK $100 (US $12.8) incentives for referrals, but there was no maximum number of referrals.

## Results

### Participating MSM: Seeds and Alters

Between March 1, 2021, and May 12, 2021, a total of 463 MSM were recruited after removing duplicates, of whom 442 (95%) completed the questionnaire. The general characteristics of participating MSM are shown in Table 1. The median age of participants was 28 (IQR 24-33) years. With 144 seeds, the seed-to-alter ratio was 1:2.07. Alters were younger (median 29 vs 31 years, *P*=.02), less likely to have used PrEP before (odds ratio [OR] 0.10, 95% CI 0.05-0.19, *P*<.001), had fewer newly acquired (*P*=.004) and previously met (*P*=.009) sex partners, and had a higher condom use rate with newly acquired male sex partners (OR 0.46, 95% CI 0.30-0.69, *P*=.009). The alters were less likely to have engaged in chemsex in the preceding year (OR 0.31, 95% CI 0.15-0.62, *P*<.001), perceive a high risk of acquiring an STI (OR 0.42, 95% CI 0.21-0.83, *P*=.01), and have previously been tested for HIV (OR 0.56, 95% CI 0.33-0.94, *P*=.03). They were more likely to have no plan to get tested if not invited by their peers (OR 1.67, 95% CI 1.04-2.68, *P*=.03) and give a confidence score lower than 9 out of 10 on collecting sufficient blood samples (OR 1.50, 95% CI 1.01-2.24, *P*=.045).

**Table 1:** General characteristics of participants and univariable comparison between seeds and alters (N=442).

		Overall	Seeds (N=144)	Alters (N=298)	OR^a^ (95% CI)	*P* value
Ethnic Chinese, n (%)		423 (95.7)	137 (95.1)	286 (96)	0.82 (0.32-2.13)	.69
Age (years), median (IQR)		29 (25-34)	31 (26-36)	29 (25-33)	N/A^b^	.02
Attained at least postsecondary education, n (%)		385 (87.1)	123 (85.4)	262 (87.9)	0.80 (0.45-1.44)	.46
Full-time employed, n (%)		288 (65.2)	92 (63.9)	196 (65.8)	0.92 (0.61-1.40)	.70
Monthly income>HK $15,000 (US $1923), n (%)		272 (61.5)	94 (65.2)	178 (59.7)	1.27 (0.84-1.92)	.26
Ever used PrEP^c^, n (%)		59 (13.3)	46 (31.9)	13 (4.4)	10.29 (5.33-19.85)	<.001
**Sexual history in the past 1 year**						
	Sought sex partners, n (%)	394 (89.1)	131 (91)	263 (88.3)	1.34 (0.69-2.62)	.39
	Newly met male sex partners, median (IQR)	3 (1-6)	4 (2-10)	3 (1-5)	N/A	.004
	Known male sex partners, median (IQR)	1 (1-2)	1 (1-3)	1 (0-2)	N/A	.009
	Always used a condom for anal sex with newly met male sex partners (N=356), n (%)	124 (34.8)	31 (25.6)	93 (39.6)	0.53 (0.32-0.85)	.009
	Always used a condom for anal sex with known male partners (N=307), n (%)	90 (29.3)	30 (27.5)	60 (30.3)	0.87 (0.52-1.47)	.61
	Engaged in chemsex, n (%)	36 (8.1)	21 (14.6)	15 (5)	3.22 (1.61-6.46)	<.001
Never been tested for HIV, n (%)		95 (21.4)	22 (15.3)	73 (24.5)	0.56 (0.33-0.94)	.03
Last HIV test within a year (N=347), n (%)		118 (34)	59 (48.3)	59 (26.2)	2.63 (1.66-4.19)	<.001
Confidence scores of at least 9 out of 10 in having sufficient volume for blood samples, n (%)		200 (45.2)	75 (52.1)	125 (42)	1.50 (1.01-2.24)	.045
No plan to get HIV tested if not participating in this study, n (%)		121 (27.4)	30 (20.8)	91 (30.5)	0.60 (0.37-0.96)	.03
Perceived high HIV risk (vs none, low, or medium), n (%)		20 (4.5)	8 (5.6)	12 (4)	1.40 (0.56-3.51)	.47
Perceived high STI^d^ risk (vs none, low, or medium), n (%)		37 (8.4)	19 (13.2)	18 (6)	2.36 (1.20-4.66)	.01

^a^OR: odds ratio.

^b^N/A: Not applicable.

^c^PrEP: pre-exposure prophylaxis.

^d^STI: sexually transmitted infection.

### The Implementation Cascade

The main results along the implementation cascade are summarized in Multimedia Appendix 1, beginning with web-based questionnaire upon registration, requisition of HIV self-tests with or without support, followed by test result upload, then web-based training, and finally completed with peer referral.

Before requesting the test kit, two-thirds (155/239, 64.9%) of the participants expressed that no support was necessary, while over half of the rest accepted to be assisted by a staff (89/155, 57.4%) or a friend or sex partner (81/155, 52.3%). Less than half (75/155, 48.4%) accepted trained peers to provide self-test support. At test kit request, most (338/434, 77.9%) did not opt for any support, while 18.9% (82/434), 1.8% (8/434), and 1.4% (6/434) requested instant messaging, video calls, and in-person support, respectively. While all participants who requested support were invited to make an appointment with the research team, only 9, 2, and 4 did so, and subsequently, 7 (78%), 0 (0%), and 3 (75%) showed up, respectively. Those requesting support were more likely not to have self-tested before (OR 0.27, 95% CI 0.16-0.48, *P*<.001), have lower confidence score of less than 9 on a scale of 1-10 in correctly performing the self-test (OR 0.35, 95% CI 0.22-0.56, *P*=.001) and interpreting the self-test result (OR 0.35, 95% CI 0.22-0.57, *P*<.001), and give a lower score in system learnability at result upload (median 75, IQR 50-100 vs median 87.5, IQR 75-100; *P*<.001; Table 2). They preferred getting tested for HIV in community-based organizations (OR 3.11, 95% CI 1.94-4.99, *P*<.001) to performing self-tests (OR 0.45, 95% CI 0.27-0.75, *P*=.002). They considered test accuracy (OR 1.65, 95% CI 1.03-2.64, *P*=.04) and the presence of staff answering sexual health questions (OR 2.57, 95% CI 1.57-4.21, *P*=.001) important, but not privacy (OR 0.50, 95% CI 0.31-0.79, *P*=.003), when deciding where to get HIV tested. They also preferred to have support before, during, and after the self-test (*P*<.001). They were less likely to worry about embarrassment when paying for an HIV self-test product (OR 0.55, 95% CI 0.34-0.89, *P*=.01). Overall, the preferred modes of self-test support were instant messaging apps (77/155, 49.7%), in-person (4/155, 47.7%), and voice call (64/155, 41.3%), while video calls and chatbots were preferred by 7.7% (12/155) and 8.4% (13/155), respectively. Multivariable logistic regression shows that a higher confidence in performing the self-test correctly (adjusted OR [aOR] 1.26, 95% CI 1.01-1.58, *P*=.04), a higher system learnability score (aOR 1.02, 95% CI 1.00-1.03, *P*=.04), concern about privacy (aOR 2.92, 95% CI 1.46-6.07, *P*=.003), and not considering posttest support important (aOR 0.44, 95% CI 0.22-0.91, *P*=.02) were associated with not requiring any forms of support.

Peer referral formed the latter part of the cascade. Over half (216/354, 61%) of participants who uploaded the test results did initiate the referral process for enrolling peers to have HIV self-test by attempting the web-based training. Participants who attempted training were more likely to have sought sex partners in the preceding year (OR 2.20, 95% CI 1.14-4.25, *P*=.02), especially through the use of location-based networking apps (OR 2.13, 95% CI 1.31-3.49, *P*=.002), but not in local saunas (OR 0.46, 95% CI 0.24-0.87, *P*=.02) (Table 3). Although they were not inclined to request a finger-prick test (*P*=.15), they were more likely to accept it (OR 1.56, 95% CI 1.00-2.44, *P*=.049) and give a confidence score of 9 or 10 in collecting sufficient blood for the self-test (OR 1.56, 95% CI 1.01-2.42, *P*=.04). They were also more likely to return the test result within 24 hours upon receiving the test kit (OR 1.80, 95% CI 1.15-2.82, *P*=.01) and less willing to pay for it (OR 0.58, 95% CI 0.35-0.95, *P*=.03). They gave a higher score in system usability (*P*=.007) and a score of 6 or 7 in the single ease question (OR 2.11, 95% CI 1.16-3.85, *P*=.01) after uploading the self-test result. Of the 200 participants who passed the web-based training, 111 (55.5%) eventually made at least one referral. They were more likely to prefer a painless HIV self-test (OR 2.09, 95% CI 1.05-4.15, *P*=.03) and not willing to pay for an HIV self-test (OR 2.53, 95% CI 1.36-4.73, *P*=.003).

**Table 2:** Univariable analysis on the characteristics of participants who accepted oral fluid tests only, as reflected in the questionnaire.

	Applied for support upon test kit request (N=96)	Did not apply for support upon test kit request (reference) (N=338)	OR^a^ (95% CI)	*P* value
Had self-tested for HIV among testers (N=347), n (%)	24 (34.3)	177 (65.6)	0.27 (0.16-0.48)	<.001
Preferred getting HIV tests at a community-based organization, n (%)	62 (64.6)	125 (37.0)	3.11 (1.94-4.99)	<.001
Preferred getting HIV tests using self-test kits, n (%)	64 (66.7)	276 (81.7)	0.45 (0.27-0.75)	.002
Considered result accuracy important when choosing an HIV test, n (%)	39 (40.6)	99 (29.3)	1.65 (1.03-2.64)	.04
Considered having staff to answer questions important, when choosing an HIV test, n (%)	36 (37.5)	64 (18.9)	2.57 (1.57-4.21)	<.001
Considered privacy important when choosing an HIV test, n (%)	36 (37.5)	185 (54.7)	0.50 (0.31-0.79)	.003
Considered degree of pain important when choosing an HIV test, n (%)	28 (29.2)	77 (22.8)	1.40 (0.84-2.32)	.20
Considered the type of body fluid used important when choosing an HIV self-test, n (%)	39 (40.6)	153 (45.3)	0.83 (0.52-1.31)	.42
Considered potential embarrassment at payment important when choosing an HIV self-test, n (%)	30 (31.3)	153 (45.3)	0.55 (0.34-0.89)	.01
Considered availability of pretest support important when choosing an HIV self-test, n (%)	36 (37.5)	54 (16.0)	3.16 (1.90-5.23)	<.001
Considered availability of midtest support important when choosing an HIV self-test, n (%)	41 (42.7)	74 (21.9)	2.66 (1.65-4.30)	<.001
Considered availability of posttest support important when choosing an HIV self-test, n (%)	41 (42.7)	71 (21.0)	2.80 (1.73-4.54)	<.001
Confidence scores of at least 9 out of 10 in correctly performing the self-test, n (%)	46 (47.9)	244 (72.2)	0.35 (0.22-0.56)	<.001
Confidence scores of at least 9 out of 10 in interpreting the self-test result, n (%)	50 (52.1)	255 (75.4)	0.35 (0.22-0.57)	<.001
System learnability score at result upload, median (IQR)	75 (50-100)	87.5 (75-100)	N/A^b^	<.001

^a^OR: odds ratio.

^b^N/A: Not applicable.

**Table 3:** Univariable analysis on the comparison between participants who had initiated the referral process and those who had not.

	Attempted training (N=216)	Did not attempt training (reference) (N=138)	OR^a^ (95% CI)	*P* value
Seed, n (%)	75 (34.7)	35 (25.4)	1.57 (0.97-2.52)	.06
Age (years), median (IQR)	28 (24-33)	28 (24-33)	N/A^b^	.93
Sought male partners in the preceding year, n (%)	198 (91.7)	115 (83.3)	2.20 (1.14-4.25)	.02
Sought male partners in local saunas in the preceding year, n (%)	19 (8.8)	24 (17.4)	0.46 (0.24-0.87)	.02
Sought male partners through mobile apps in the preceding year, n (%)	175 (81.0)	92 (66.7)	2.13 (1.31-3.49)	.002
Never been tested for HIV before joining this study, n (%)	42 (19.4)	32 (23.2)	0.80 (0.48-1.34)	.40
Accepted finger-prick self-tests, n (%)	93 (43.1)	45 (32.6)	1.56 (1.00-2.44)	.049
Requested a finger-prick self-test, n (%)	95 (44.0)	50 (36.2)	1.38 (0.89-2.14)	.15
Confidence 9 or above out of 10 in collecting enough blood for self-test, n (%)	105 (48.6)	52 (37.7)	1.56 (1.01-2.42)	.04
Would not pay for a self-test, n (%)	70 (32.4)	30 (21.7)	1.73 (1.05-2.83)	.03
Uploaded results within 24 hours after collecting the test kit, n (%)	110 (56.1)	54 (41.5)	1.80 (1.15-2.82)	.01
Learnability score at result upload, median (IQR)	87.5 (75-100)	88 (63-100)	N/A	.11
Usability score at result upload, median (IQR)	81 (72-94)	75 (66-88)	N/A	.003
Single-ease question scores 6 or above at result upload, n (%)	191 (89.3)	110 (79.7)	2.11 (1.16-3.85)	.01

^a^OR odds ratio.

^b^N/A: Not applicable.

Of 354 (81.6%, N=434) results returned, 333 (94.1%) were negative, 4 (1.1%) were positive, and 17 (4.8%) were invalid. Confining to negative and positive results only, compared with research team’s interpretation, the accuracy of participant’s result interpretation was 99.1% (95% CI 97.4-99.8). Of the positive screening test results, 2 were confirmed to be true positives, including one who had already been on antiretroviral treatment. The newly diagnosed subject had been referred to HIV services with consent. Participants who did not upload their self-test results had a higher monthly income (OR 1.95, 95% CI 1.13-3.35, *P*=.01), a lower condom use rate with newly met male sex partners in the preceding year (OR 0.50, 95% CI 0.27-0.93, *P*=.03), and preferred self-testing (OR 2.18, 95% CI 1.07-4.41, *P*=.03) to undergoing testing at community-based organizations (OR 0.50, 95% CI 0.30-0.85, *P*=.009). Alters who uploaded the test results, compared with those who did not, were more concerned about the accuracy of HIV test (OR 2.49, 95% CI 1.15-5.38, *P*=.02) and delivery mode of the self-test (OR 2.16, 95% CI 1.12-4.19, *P*=.02), and preferred to receive an HIV test at community-based organizations (OR 2.28, 95% CI 1.15-4.53, *P*=.02).

### Preferred Modes of HIV Self-testing

Given the same level of accuracy, more than a half (261/442, 59.0%) preferred oral fluid self-tests, 23.3% (103/442) preferred finger-prick self-test, and 17.6% (78/442) showed no preference. Participants who accepted finger-prick tests were more likely to have a lower income (OR 1.51, 95% CI 1.02-2.24, *P*=.005), give at least a 9 in confidence score in collecting sufficient blood samples for self-test (OR 3.08, 95% CI 2.07-4.58, *P*<.001), and eventually request a finger-prick test (OR 34.34, 95% CI 20.02-58.89, *P*<.001). They preferred getting tested in a community-based organization (OR 1.50, 95% CI 1.02-2.20, *P*=.04) rather than performing self-tests (OR 0.50, 95% CI 0.32-0.79, *P*=.003). When choosing the place for an HIV test, those who accepted oral fluid tests only were more likely to consider pain (*P*<.001) and the presence of a staff for answering sexual health questions (*P*=.03) important (Table 4). They were also concerned about the type of body fluid used for a self-test (*P*<.001) and potential embarrassment when paying for a self-test product at the cashier (*P*=.02). On the other hand, those who requested a finger-prick test were more likely to give a higher confidence score in collecting sufficient blood for the self-test (aOR 1.42, 95% CI 1.27-1.61, *P*<.001), and to consider pain unimportant when choosing an HIV test (aOR 0.37, 95% CI 0.18-0.72, *P*=.004).

**Table 4:** Univariable analysis on the characteristics of participants who had applied for support at test-kit request.

	Accepted finger-prick tests (N=181)	Accepted oral fluid tests only (reference) (N=261)	OR^a^ (95% CI)	*P* value
Had HIV self-tested among testers (N=347), n (%)	124 (61.1)	81 (56.3)	1.22 (0.79-1.88)	.37
Preferred getting HIV tests at a community-based organization, n (%)	88 (48.6)	101 (38.7)	1.50 (1.02-2.20)	.04
Preferred getting HIV tests using self-test kits, n (%)	129 (71.3)	217 (83.1)	0.50 (0.32-0.79)	.003
Considered result accuracy important when choosing an HIV test, n (%)	59 (32.6)	81 (31)	1.07 (0.72-1.61)	.73
Considered having staff to answer questions important when choosing an HIV test, n (%)	32 (17.7)	69 (26.4)	0.60 (0.37-0.96)	.03
Considered privacy important when choosing an HIV test, n (%)	85 (47)	140 (53.6)	0.77 (0.52-1.12)	.17
Considered degree of pain important when choosing an HIV test, n (%)	22 (12.2)	87 (33.3)	0.28 (0.17-0.46)	<.001
Considered the type of body fluid used important, when choosing an HIV self-test, n (%)	50 (27.6)	148 (56.7)	0.29 (0.19-0.44)	<.001
Considered potential embarrassment at payment important when choosing an HIV self-test, n (%)	65 (35.9)	123 (47.1)	0.63 (0.43-0.93)	.02
Considered availability of pretest support important when choosing an HIV self-test, n (%)	37 (20.4)	57 (21.8)	0.92 (0.58-1.46)	.72
Considered availability of midtest support important when choosing an HIV self-test, n (%)	45 (24.9)	73 (28)	0.85 (0.55-1.31)	.47
Considered availability of posttest support important when choosing an HIV self-test, n (%)	48 (26.5)	69 (26.4)	1 (0.65-1.54)	.98
Confidence scores of at least 9 out of 10 in correctly performing the self-test, n (%)	125 (69.1)	170 (65.1)	1.19 (0.80-1.79)	.39
Confidence scores of at least 9 out of 10 in interpreting the self-test result, n (%)	127 (70.2)	183 (70.1)	1 (0.66-1.52)	.99
System learnability score at result upload, median (IQR)	75 (62.5-100)	87.5 (75-100)	—^b^	.08

^a^OR: odds ratio.

^b^—: Not available.

## Discussion

### Overview

The study results showed that leveraging seed participants’ social network was a feasible approach to recruit harder-to-reach members in the MSM community who have never been tested for HIV with the use of HIV self-testing. A systematic review showed that such an approach was feasible and could enhance the acceptability of HIV services and facilitate the diagnostic process [23]. Alters recruited by the seed participants in our study had a suboptimal condom use rate, but they did not perceive having a high risk for STI. They were also more likely to be non–HIV-testers and would likely remain to be untested if not engaged by the seeds participating in this study, echoing results from a previous study [24]. These observations highlighted the importance of enrolling and training appropriate seeds who would be best positioned to identify MSM requiring HIV tests in implementing a social network–based approach for broadening the catchment of self-testing in MSM [25].

Successful implementation of network-based HIV testing hinges on the functioning of an effective referral program that promotes the involvement of motivated peers. Programs providing appropriate training to community members for distributing HIV self-tests were shown to be effective [25-27]. Our study showed that those who initiated the referral process by completing the web-based training had good connections with other community members. They were confident in and accepted the use of blood-based self-tests and could therefore serve as the point of reference when their peers encountered problems performing self-tests [28]. On the other hand, the positive feedback illustrated by their high system usability scores incited them to refer their peers [29]. Comparatively, those who were less confident to perform the self-test were less willing to invite their peers. In fact, having confident and satisfied users to recruit subjects can be beneficial to the alters as they have the experience, knowledge, and relationship with the peer to provide necessary support.

Our cascade analyses showed that when operating a self-test program, support services should be in place in response to the users’ needs, especially for those who had never self-tested and were unconfident in performing self-tests. From the user’s perspective, lacking real-time support may hinder their confidence in performing the self-test and result in errors [30]. Appropriately tailored counseling services and follow-ups are necessary, but they are not always provided concurrently [31]. From the health service providers’ perspective, linkage to care is crucial to provide confirmation tests and have treatment initiated after diagnosis, but these rely on the voluntary submission of self-test results, for which a suitable platform should be established [14]. Nevertheless, self-tests offer an acceptable alternative means for MSM who prefer testing at home, thus enabling access to be extended to otherwise harder-to-reach community members [32]. A general approach for promoting and distributing self-tests can be a web-based platform from which MSM could take the initiative to acquire the tests [33]. Alternatively, this can be a networked program with members or influencers who can personally identify and provide support to social network members who would likely benefit from an HIV self-test [18,32]. In our study, those requiring support needed someone to answer HIV-related questions and were concerned about the self-test’s accuracy. Practically, some MSM would need support for performing the self-test through digital contacts with persons rather than chatbots. The participants in this study accepted and preferred digital means for receiving supports for HIV self-test, echoing the results from a systematic review [34]. In particular, web-based interventions have shown high acceptability and usability in previous Chinese studies; supporting personnel giving instructions and real-time guidance through the HIV self-test process is one of the acceptable means for promoting HIV self-testing. Our study result, despite the small number of participants requesting support, also coincided with the review’s finding that text-based support is preferred over video-based one. Contrarily, those needing no support preferred privacy-preserving self-tests which allowed them to get tested without someone scrutinizing their sexual activities in the conventional voluntary counseling and testing process [35], signifying that both community-based services and self-tests should coexist as they suit different people’s needs.

Preferences for specific type of self-tests constituted an important consideration in the implementation of self-testing. Noninvasive self-tests were generally more welcomed by MSM in Hong Kong, particularly by those who refused to attend the service of community-based organizations, were afraid of pain, and were not confident in self-collecting sufficient blood samples. However, in a South African study, the opposite was found, with more MSM participants trusting blood-based rather than oral fluid [36]. These findings suggested that local beliefs and preferences could vary by cultural and social environments.

Finally, linkage to care for HIV positive MSM is an integral component of an HIV self-test program, which primarily relies on voluntary submission of test results. A previous study showed that only half of the participants were willing to share their results with researchers or health care workers [37]. The return rate in our study was relatively high (354/434, 82%), which may be related to the provision of support in the cascade. Those who did not return the result actually preferred self-testing to community-based service but were apparently unconcerned about the delivery modes or types of body fluid used. As they also had a lower condom use rate, it could be worrying if those who self-tested positive did not seek follow-up services, not least a confirmation test, as self-tests only play the screening role. Although some participants decided not to return their results, a meta-analysis showed that HIV self-tests do not necessarily reduce the rate of linkage to care or treatment [38]. Alters returning results were concerned about the self-test’s accuracy; therefore, they may want their results to be confirmed by the provider. This also explained their preference for getting tested at a community-based organization where instant support from the staff or volunteer would be available and they would be free from the pressure to collect sufficient blood sample for the test and to interpret the result on their own, fearing that the interpretation could be wrong. Both the suboptimal sensitivity of user result interpretation and the imperfect result return rate prompted a need for a real-time tool for self-test result collection and interpretation.

This study carries several limitations. Unlike some other HIV self-test studies, we have not adopted a deposit system to encourage participants to return their test results; otherwise, the return rate would be even higher. The logistics of including deposit in a web-based distribution system normally involves mobile payment services, which requires the use of a credit card [39], which may not be possessed by everyone and concerns about privacy may arise. To eliminate potential access barriers, our program has relied on the issuance of reminders by referrer, as the latter could only collect the incentives upon the alter’s upload of results. While the incentives may not be attractive enough to achieve this purpose, the mechanism was more ethically appropriate which did not coerce the referrer. The drawback of not having all results returned was that we could not monitor all results, although failure of returning result could be a sign of refusing follow-ups. The referrer and the study website could be two of the resources a participant relied on, but we have not collected such information. Separately, as the study was time-limited, participants recruited toward the end of the study period may not have sufficient time to make a successful referral, although at least 7 days had been given for uploading test results after delivery of the kits. Although we allowed additional time for participants recruited later in the accrual period to make referrals, the reasons why participants were not interested in making referrals were not collected. In the analysis, we used the initiation of referral process instead of actual successful referral to alleviate this bias and characterize participants who were willing to refer their peers. Similar to other cross-sectional studies in the field, social desirability and recall biases may occur with the sensitive nature of the questionnaires. We adopted a self-administered approach and used a relatively short recall period to minimize such biases. Like other community-based studies, self-selection bias may occur; especially those who were more concerned about their sexual health may be more likely to have joined this study.

### Conclusions

Our study has shown that the social network approach was effective in reaching MSM especially those who had not been HIV tested and even had no plan for an HIV test. Both community-based testing services and self-tests were acceptable to MSM with different considerations; and the 2 types of HIV self-tests, namely blood-based and oral fluid–based, were preferentially chosen by similar proportions of participants with unique characteristics, such as confidence in collecting blood samples and fear of pain. Self-testing is a multistep process, which could be challenging for MSM unfamiliar with parts of the procedure. In the implementation of an HIV self-testing program, live support by real persons, regardless by digital means or physical appearances, is imperative in ensuring the tests have been performed properly at the community level. An implementation cascade–based evaluation could be invaluable for evaluating the performance of such program. To reach the UNAIDS fast-track targets, multiple HIV testing options should be made accessible, and MSM peer engagement can play a paramount role in its promotion.

## Appendix

Multimedia Appendix 1 Implementation cascade of a social network–based HIV self-test trial.

## Abbreviations

aOR: adjusted odds ratio
MSM: men who have sex with men
OR: odds ratio
PrEP: pre-exposure prophylaxis
STI: sexually transmitted infection
UNAIDS: Joint United Nations Programme on HIV/AIDS

## Data Availability

The data sets generated and analyzed during this study are available from the corresponding author on reasonable request and approval from the Chinese University of Hong Kong—New Territories East Cluster Clinical Research Ethics Committee.
