# Supplementary figures and images for: Implementation Cascade of a Social Network–Based HIV Self-testing Approach for Men Who Have Sex With Men: Cross-sectional Study
Source: J Med Internet Res. 2023 Apr 26;25:e46514. doi: 10.2196/46514 (PMC10173037; doi:10.2196/46514)

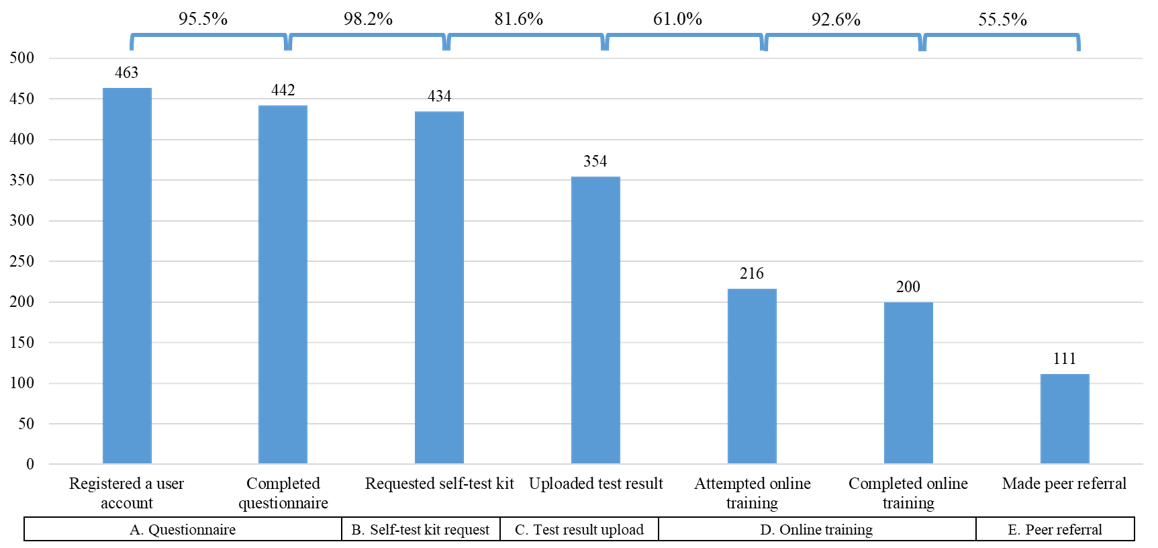

Supplement: Multimedia Appendix 1 [file jmir_v25i1e46514_app1.png]
